# Analysis of deep sequencing microRNA expression profile from human embryonic stem cells derived mesenchymal stem cells reveals possible role of let-7 microRNA family in downstream targeting of Hepatic Nuclear Factor 4 Alpha

**DOI:** 10.1186/1471-2164-11-S1-S6

**Published:** 2010-02-10

**Authors:** Winston Koh, Chen Tian Sheng, Betty Tan, Qian Yi Lee, Vladimir Kuznetsov, Lim Sai Kiang, Vivek Tanavde

**Affiliations:** 1 Bioinformatics Institute (BII), Agency of Science Technology and Research (A*STAR) 30 Biopolis Street, #07-01 Matrix, Singapore 138671; 2 Institute of Medical Biology A*STAR 8A Biomedical Grove, #06-06 Immunos, Singapore 138648

## Abstract

**Background:**

Recent literature has revealed that genetic exchange of microRNA between cells can be essential for cell-cell communication, tissue-specificity and developmental processes. In stem cells, as in other cells, this can be accomplished through microvesicles or exosome mediated transfer. However, molecular profiles and functions of microRNAs within the cells and in their exosomes are poorly studied. Next generation sequencing technologies could provide a broad-spectrum of microRNAs and their expression and identify possible microRNA targets. In this work, we performed deep sequencing of microRNAs to understand the profile and expression of the microRNAs in microvesicles and intracellular environment of human embryonic stem cells derived mesenchymal stem cells (hES-MSC).

We outline a workflow pertaining to visualizing, statistical analysis and interpreting deep sequencing data of known intracellular and extracellular microRNAs from hES-MSC). We utilized these results of which directed our attention towards establishing hepatic nuclear factor 4 alpha (HNF4A) as a downstream target of let-7 family of microRNAs.

**Results:**

In our study, significant differences in expression profile of microRNAs were found in the intracellular and extracellular environment of hES-MSC. However, a high level of let-7 family of microRNAs is predominant in both intra- and extra- cellular samples of hES-MSC. Further results derived from visualization of our alignment data and network analysis showed that let-7 family microRNAs could affect the downstream target HNF4A, which is a known endodermal differentiation marker. The elevated presence of let-7 microRNA in both intracellular and extra cellular environment further suggests a possible intercellular signalling mechanism through microvesicles transfer. We suggest that let-7 family microRNAs might play a signalling role via such a mechanism amongst populations of stem cells in maintaining self renewal property by suppressing HNF4A expression. This is in line with recent paradigm where microRNAs regulate self-renewal and differentiation pathways of embryonic stem cells by forming an integral biological network with transcription factors.

**Conclusion:**

In summary, our study using a combination of alignment, statistical and network analysis tools to examine deep sequencing data of microRNAs in hES-MSC has led to a result that (i) identifies intracellular and exosome microRNA expression profiles of hES-MSCwith a possible mechanism of miRNA mediated intercellular regulation by these cells and (ii) placed HNF4A within the cross roads of regulation by the let-7 family of microRNAs.

## Background

Small RNAs play a wide range of regulatory roles from degradation to translational silencing of messenger RNA. The most studied class of small regulatory RNA is microRNAs (miRNAs). miRNAs are about 22 nucleotides long and have been identified in animals, plants and viruses. Precursor miRNAs have stem loop structures that are cleaved by Drosha and Dicer forming mature functional miRNA molecule. By forming RNA-induced silencing complexes, miRNA can either cleave messenger RNA molecules or inhibit translation. Through such mechanisms, they are involved in various cellular processes including hematopoietic differentiation and cell cycle regulation [1,2].

The amount of genetic information regulated post-transcriptionally by miRNAs is potentially huge. Computational and indirect evidences indicate that miRNAs might regulate up to a third of all genes making direct and indirect consequences of miRNA directed regulation significant [3]. The resulting regulatory network is very often an extensive and complex one.

Deep sequencing provides a rapid and sensitive way of obtaining miRNA profiles expressed by human embryonic derived mesenchymal stem cells (hES-MSC). These cells have the ability to differentiate into multiple mesenchymal phenotypes, such as bone, cartilage, tendon and adipose tissue [4]. This property with a broad distribution of sources makes MSC an attractive therapeutic target.

Despite this level of interest, a clear understanding of the factors involved in regulation of MSC remains rudimentary. Global gene expression analysis has revealed that MSC differentiation into specific mature cells types is a temporally controlled and regulated process [5,6]. miRNAs provide an attractive mechanism for temporal regulation of mRNA translation and stability. Regulation of miRNA expression pattern can then be perceived as a novel regulatory network affecting cellular function. Organization of the hierarchical order of stem cell types based on the linkage of their functional characteristics to such regulatory elements might present a novel means to understand and eventually manipulate cell fate. This miRNA mediated regulation of stem cell differentiation however will act in concert with other methods of regulation of gene expression like transcription factors, epigenetic mechanisms etc. Therefore it is important to interpret the results of this study in the context of understanding miRNA regulation in concert with other regulatory mechanisms that control cellular differentiation.

Recent literature has also revealed that genetic exchange of mRNA and miRNA between cells can be accomplished through microvesicles or exosome mediated transfer [7]. Microvesicles can be shed from surfaces of activated cells or derived from the endosomal membrane compartment after fusion of secretory granules with the plasma membrane where they exist as intraluminal membrane-bound vesicles. Embryonic stem cells are a source of such microvesicles. It is possible that embryonic stem cell derived microvesicles contain biologically active molecules that affect growth and cell fate decision of targeted cells. Similar vesicles released from human and murine mast cell lines were shown to contain miRNAs [8]. Employing microvesicles for transfer of genetic material would be an efficient means for these intercellular communications [9]. It is conceivable that MSC derived from embryonic stem cells also communicate with neighbouring cells using microvesicles. In this study, we performed deep sequencing of small RNAs to understand the expression of miRNAs in both microvesicles and intracellular environment of hES-MSC.

This convergence of deep sequencing technology and the potential regulatory roles of miRNA in stem cells provide many opportunities as well as challenges in terms of data mining methodology in uncovering biological significance. Bioinformatics tools are necessary to bridge the gap between raw sequencing data and biological significance of these regulatory RNAs. A fundamental difficulty lies in identifying miRNA targets as well as deciphering the exact mechanism of translational repression of mRNAs by these snippets. Considerable work has been put into computational prediction of miRNA targets. Many challenges still remain in integrating large sequencing datasets to obtain gene-level distinctions.

In our study, we present a novel workflow using existing tools available for understanding these sequencing data sets. Our workflow extracts salient features from sequencing data for visualization and focuses on potential relationships between miRNAs with gene targets. Applying our workflow for quantitative estimation of small RNAs in hES-MSC intra and extra cellular examples, we can develop a better understanding of exosome mediated miRNA involvement in the regulation of other cells in the hES-MSC microenvironment.

## Results

### Distribution of miRNAs from Deep Sequencing showed distinct phases

**Figure 1:**
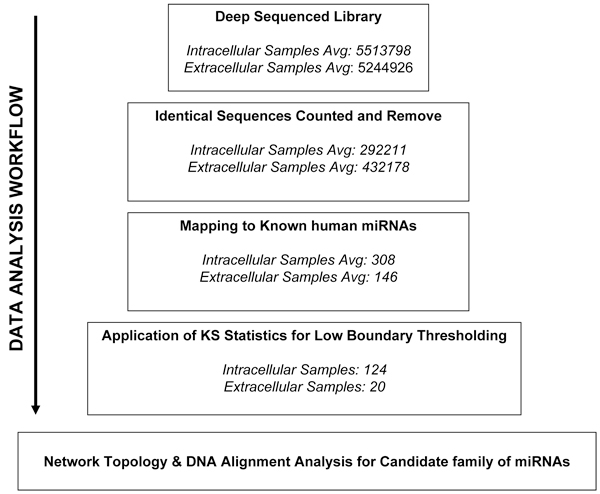
**Different stages of processing microRNA sequences to gain biological insight**. Massive sequencing data can be overwhelming to examine, with each stage of processing choices are made to direct analysis towards biological significance and understanding. Initial processing stages emphases on visualization of datasets in different forms such as annotations and alignment in the genome. This is to allow for quick assessment of salient features. Following this are more advanced analysis exploiting existing tools for examining distributions and networks topology to conjure biological hypothesis for verification. The entire workflow can then be thought as a funneling process towards biologically interesting interactions for verification in our hES-MSC cell line.

Massive sequencing data can be overwhelming to examine. With each stage of processing, choices are made to direct analysis towards biological significance and understanding. Initial processing stages emphasize on visualisation of datasets in different forms such as annotations and alignment in the genome. This allows for quick assesment of salient features. Following this are more advanced analyses exploiting existing tools for examining distrbutions and networks topology used to conjure biological hypothesis for verification. The entire workflow can then be thought as a funneling process towards identifying biologically interesting interactions of miRNAs with genes in hES-MSC (Figure 1). These interactions can then be experimentally verified.

**Figure 2:**
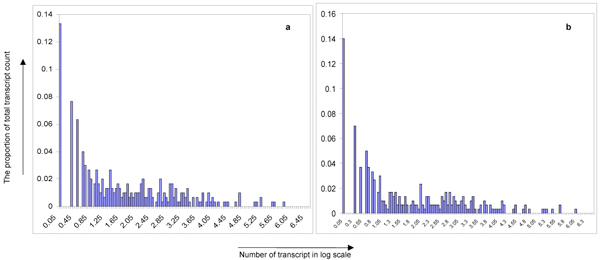
**High abundant expression signals of detected miRNAs in two independent replicates show similar frequency distributions**. Distributions of the expression signal count (y-axis: representing the proportion of total transcript counted and the x-axis: representing the count of signals; data in a log scale) derived from biological replicates of intracellular samples show a high degree of similarity. The empirical distributions can be interpreted as a mixture of two essentially distinct distributions. The low-abundant count (presented on the left part of the distribution) provides noise-rich data. This data shows an exponential decline of the frequency distribution suggesting that a large portion of signal have low counts. This is followed by a fairly even distribution across the log scale of counts and ended with sporadic signal that have high counts at the far end of the log scale. The high-abundant count (presented on the right part of the distribution) has a long tail and shows log-normal-like frequency distribution. The last part of the distribution shows high level of similarity in the right-size trends, which is is exploited in statistical tests for determining significances/reliable signals from mostly noise signals.

One of the main challenges in interpreting deep sequencing data is the large number of transcripts with small read counts in the range of 1 to 10. Therefore there is a need to determine a threshold value for sieving out significant miRNAs. We address this issue by first examining the overall distribution of transcripts in our samples which are biological replicates. Plots were made to interpret the global expression distribution for these miRNAs as depicted in Figure 2. Such plots can be interpreted to give the probability of finding a particular miRNA given an associated count/abundance.

**Figure 3:**
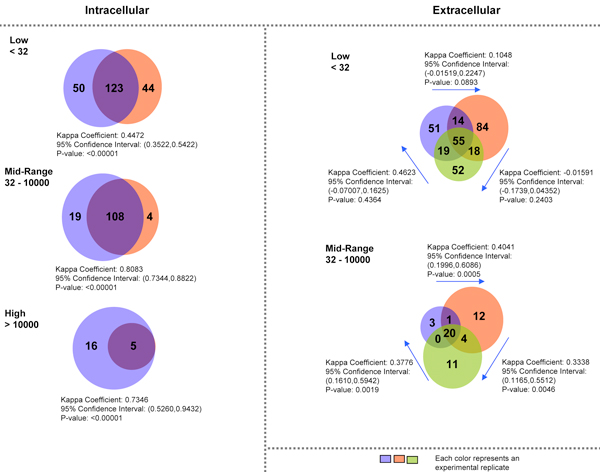
**Venn Diagrams depict the difference of miRNA transcript counts identified in intracellular and extracellular (secreted) miRNA sets**. Due to statistical results presented on Figure 3, the transcript count domains are broadly separated into 3 main levels: Low (below 32 transcripts), Mid-range (32-10000) and High (>10000). Within each sub-interval of the miRNA expression value, Venn diagrams are used to analysis the degree of overlapping between experimental replicates. Kappa correlation coefficient, reflecting the degree of agreement between two sets (Cytel Studio@7) was calculated for each pair of replicates. In general, for intracellular compartment miRNAs the degree of overlapping (agreement between experiments) for the Low category is much lower compared to the mid-range and high data subset. This result suggests a good experimental responsibility of occurrence of miRNAs expressed at the moderate- and high-abundant expression levels. The low-abundant set of miRNA shows poor reproducibility, perhaps due to experimental and biological noise. For the extracellular miRNA samples, there were no transcripts at the high expression category. Moreover, a low probability of co-occurrence of the same miRNAs (non-significant kappa correlation) in low expression category or in moderate expression category is found.

**Figure 4:**
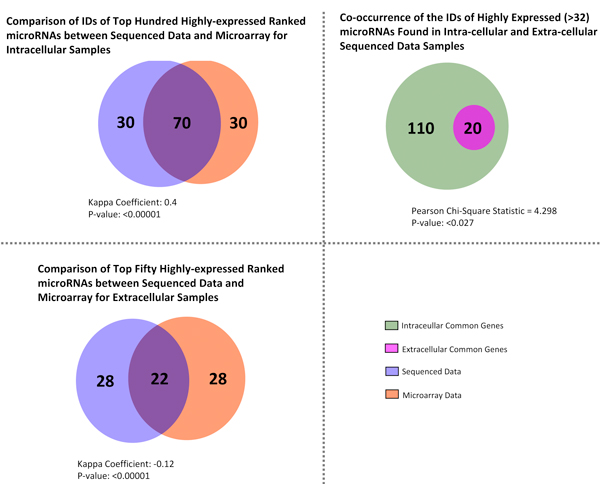
**Additional Venn diagram analysis**. A) Considerable overlap in top ranked genes from comparing of highly-expressed microarray and sequencing data for intracellular miRNAs. B) Overlap in top ranked genes from comparing of highly-expressed microarray and sequencing data for extracellular miRNAs is low. C) Top-level sequence data shows strong agreement between intra and extra cellular samples. Expression microarray for miRNAs were performed and compared with our deep sequencing data. Ranking of the top miRNAs by their abundance from both techniques was performed. Top hundred miRNAs for the intracellular samples were compared using kappa correlation analysis and a large degree of overlap between studied sets was observed. Similarly for the extra cellular samples, the top 50 miRNAs were compared. The degree of overlap between studied sets was less than that of the intracellular samples. A final comparison involves that of the common genes amongst replicates of the intracellular samples with the extra cellular samples. In this case we found that most of the genes of the extracellular microRNA form a common subset of the genes of the intracellular miRNA.

In general, the distribution shows three distinct phases. A large number of unique transcripts have low count number and are distributed unevenly across the low count number range. This is followed closely by a subsequent phase where the numbers of unique transcripts are distributed evenly across a large range of count number magnitude. The last phase follows the previous with transcripts reverting to an uneven distribution of high count number. These phases can be categorised into different groups based on transcript counts. Figures 3 and 4 show this classification which reflects the degree of agreement and overlap in each case.

Considering the initial uneven distribution of transcripts with low count number, it is a possibility that this large group of transcripts constitute a form of noise since this group of transcripts is expressed sporadically with low counts. The concept of noise here is distinct from noise arising from stochastic variations in the expression level of a given gene in individual cells. It is possible that these transcripts arose from imperfect fidelity of Pol II transcriptional machinery due to stochastic components governing fundamental interactions between molecules within the cell. Alternatively, it could also be an artefact inherent to sequencing.

Transcripts of the subsequent phase with even distribution can then be inferred to be another group of miRNAs that constitute a steady state expression of miRNA genes in MSC followed by the last phase consisting of miRNAs with large count numbers that contributes significantly to the unique phenotype exhibited by such cells. From a genome wide perspective, this representation and interpretation of data presents a possibility to distinguish the relative proportions of biologically significant miRNAs from potential noise. To corroborate such a view with a quantitative aspect, a statistical test based strategy was developed.

### Statistical Method employed implies transcript count of 32 as the minimum threshold

**Figure 5:**
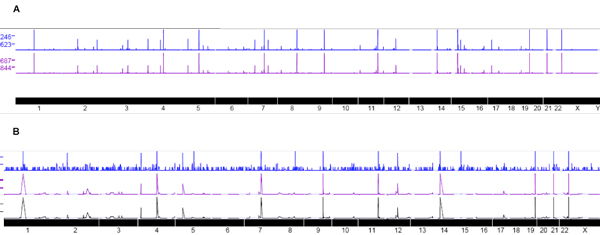
**Different hES-MSC replicates show similar alignment peaks**. Visualization of Seqmap mapping via UCSC genome browser reveals peaks along specific genomic regions that have large numbers of miRNA binding to these regions. In the top figure A Blue and Purple trend lines represent biological replicates of samples derived from intracellular environment. High degree of correlation is observed between the replicates as can be observed from the similarity in locations where peaks were found. The height of each such peak corresponds to the number of transcripts detected from deep sequencing. Each peak now represents genomic locations where a large number of specific transcripts bind to. The bottom figure B depicts the extra cellular sample transcripts that are aligned to the human genome using Seqmap. Peaks occur in similar region after mapping across the replicates. Each replicate is visualized with a different color and the salient feature reveals peaks from different sample aligning in similar locations.

**Figure 6:**
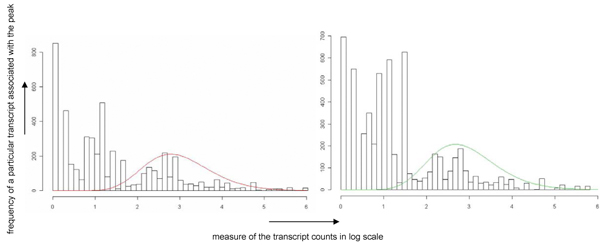
**Histograms of peak magnitudes show a similar distribution after an initial uneven trend**. Distributions of peak magnitudes [Y-axis shows the frequency of a particular transcript associated with the peak occurring; x-axis is a measure of the transcript counts in log scale] after mapping shows the same trend after an initial uneven distribution. Green and red trend lines are least square fits of gamma distribution function reflecting that both samples showed great similarity after the initial uneven distribution possibly suggesting that the initial phase to be noise.

**Figure 7:**
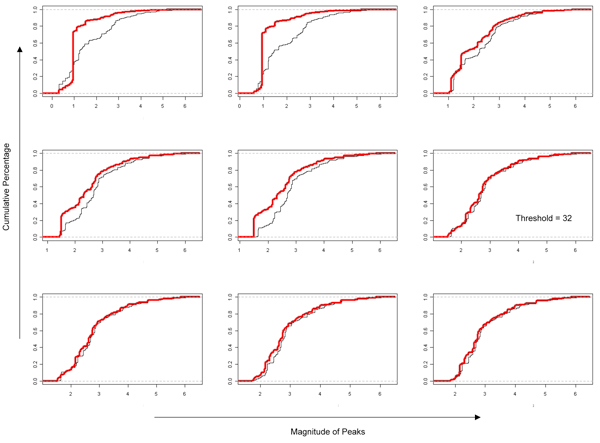
**32 transcripts is an optimum threshold for achieving similar cumulative frequency distribution amongst replicates**. KS statistics was applied iteratively to our biological replicates (red and black lines), each graph depicts a gradual change in the threshold value. The KS test statistics can be thought of as a cost function that we seek to minimize to ensure that the distributions between the two replicates are similar. Applying such a strategy iteratively as we change the threshold value gradually, we arrive at a point where both line converge together indicating similar distributions. The point where this first occurs is designated as the minimum threshold count value of biological significance.

To achieve this quantitative threshold value for further analysis, we began by mapping reads to the human genome. Seqmap was used to map known human miRNA transcript sequence data with 3 mis-matches in alignments to the human genome. The resulting genomic graphs (Figure 5) combined with read counts when observed in the UCSC genome browser reveals peaks along genomic locations. Histograms of the peaks magnitude distribution (Figure 6) reveal that, after an initial transient uneven distribution phase, peaks are similar amongst our biological replicates. To achieve a threshold value, an adaptive thresholding method using KS statistics is deployed (Figure 7). This value came to a read count of 32. Transcripts with a read count of more than 32 are thus deemed to be significantly different from noise. This threshold value of 32 read counts coincide with the point of inflexion in the distributions of Figure 2 that marks the separation of the initial transient uneven phase with the later stable phase.

### High read counts of let-7 family miRNAs transcripts are present in both intra and extra cellular samples of hES-MSC

Using the 32 read counts as the threshold, ranking according to read counts was performed on this subset of human miRNAs. Table 1 shows the highest ranking miRNA transcripts in the intracellular space, whereas Table 2 lists the highest ranking miRNA transcripts in the extracellular space. The complete list of miRNAs in the intracellular and extracellular space of MSC ranked according to their abundance is given in supplementary file 1. let-7 family of miRNAs is represented predominantly in the top rankings miRNAs in both intra and extra cellular samples of hES-MSC. let-7 family of miRNAs was first identified in C. elegans and has since been emerging as having important tumour-supressor role. Moreover, it also marks a temporally controlled switch from stem cells to differentiated cell fate. This led to our growing interest of let-7 miRNA's functional roles in hES-MSC. Apart from the let-7 family, other miRNAs like miR199b, miR22 & miR143 were also significantly overrepresented in both the intracellular and the extracellular hES-MSC samples. It is also interesting to note that there is significant overlap between the lists of miRNA represented in the intracellular and extracellular space. We were unable to find any miRNAs that were overrepresented only in the extracellular space and not in the intracellular hES-MSC space.

**Table 1:** Let 7 family of miRNAs are expressed strongly by hES-MSC within the cells. This table lists common transcripts in the intracellular samples in the high category of more than 10,000 transcripts showing a predominance of let 7 family of miRNAs.

Intracellular Transcripts Number (Sample 2)	Intracellular Transcripts Number (Sample 1)	Transcript Sequence	miRNA Annotation
**1076532**	**667868**	**TGAGGTAGTAGATTGTATAGTT**	**hsa-let-7f**
**425364**	**363558**	**TGAGGTAGTAGTTTGTACAGTT**	**hsa-let-7g**
406433	342886	ACAGTAGTCTGCACATTGGTTA	hsa-miR-199a-3p
179887	169479	TAGCACCATCTGAAATCGGTTA	hsa-miR-29a
**256612**	**164108**	**TGAGGTAGTAGGTTGTATAGTT**	**hsa-let-7a**
**162041**	**133623**	**TGAGGTAGTAGTTTGTGCTGTT**	**hsa-let-7i**
145189	114380	TAGCTTATCAGACTGATGTTGA	hsa-miR-21
51885	49588	TCAGTGCATGACAGAACTTGG	hsa-miR-152
49042	48131	TGAGATGAAGCACTGTAGCTC	hsa-miR-143
67564	46368	AGCTACATTGTCTGCTGGGTTTC	hsa-miR-221
52911	39313	AGCAGCATTGTACAGGGCTATGA	hsa-miR-103
28369	23084	AACCCGTAGATCCGAACTTGTG	hsa-miR-100
**26641**	**21523**	**TGAGGTAGTAGGTTGTATGGTT**	**hsa-let-7c**
**29311**	**19314**	**TGAGGTAGGAGGTTGTATAGTT**	**hsa-let-7e**
15299	13189	TGGCTCAGTTCAGCAGGAACAG	hsa-miR-24
13269	10037	TCCCTGAGACCCTAACTTGTGA	hsa-miR-125b

**Table 2:** Let 7 family of miRNAs are secreted by hES-MSC in large numbers. This table lists common transcripts in the extracellular samples that belong to the mid-range category of between 32 to 10,000 transcripts showing a predominance of let 7 family of miRNAs. It is interesting to note that no transcripts with counts greater than 10,000 were observed in the extracellular space.

Extracellular Transcript Number (Sample 1)	Extracellular Transcript Number (Sample 2)	Extracellular Transcript Number Sample 3	Transcript Sequence	miRNA Annotation
**826**	**3653**	**3734**	**TGAGGTAGTAGATTGTATAGTT**	**hsa-let-7f**
**679**	**2748**	**1870**	**TGAGGTAGTAGTTTGTACAGTT**	**hsa-let-7g**
287	69	331	AAGCTGCCAGTTGAAGAACTGT	hsa-miR-22
254	992	249	TGGAATGTAAAGAAGTATGTAT	hsa-miR-1
243	605	997	ACAGTAGTCTGCACATTGGTTA	hsa-miR-199a-3p
**215**	**1225**	**931**	**TGAGGTAGTAGGTTGTATAGTT**	**hsa-let-7a**
182	227	644	TAGCTTATCAGACTGATGTTGA	hsa-miR-21
170	100	515	TCAGTGCATGACAGAACTTGG	hsa-miR-152
164	389	834	TAGCACCATCTGAAATCGGTTA	hsa-miR-29a
112	42	160	TCCCTGAGACCCTAACTTGTGA	hsa-miR-125b
**95**	**1051**	**149**	**TGAGGTAGTAGGTTGTATGGTT**	**hsa-let-7c**
94	40	156	TGGCTCAGTTCAGCAGGAACAG	hsa-miR-24
87	159	100	AAAAGCTGGGTTGAGAGGGCGA	hsa-miR-320a
**82**	**308**	**334**	**TGAGGTAGTAGTTTGTGCTGTT**	**hsa-let-7i**
81	97	43	TGAGGGGCAGAGAGCGAGACTTT	hsa-miR-423-5p
71	55	310	TGGAGAGAAAGGCAGTTCCTGA	hsa-miR-185
66	46	256	AACCCGTAGATCCGAACTTGTG	hsa-miR-100
66	502	277	TGAGATGAAGCACTGTAGCTC	hsa-miR-143
**39**	**104**	**87**	**AGAGGTAGTAGGTTGCATAGTT**	**hsa-let-7d**
39	111	194	AGCTACATTGTCTGCTGGGTTTC	hsa-miR-221

### Complexity Reduction using Gene interaction Networks revealed similarity in topology that suggested downstream targets for let-7 family of miRNAs

miRNAs are known to down regulate gene expression. This can be done by direct mRNA cleavage, mRNA decay by deadenylation or translational repression. The greatest challenge besetting incorporation of miRNA regulation into known gene expression mechanism is the great difficulty in predicting mRNA targets of miRNAs. Although the binding event behind the translational repression and mRNA degradation is driven primarily by complementarity between miRNA and target sites, computationally predicted target interactions generally generate a large list of targets. One such prediction algorithm, TargetScan requires perfect complementarity to the seed region of miRNA and extends these regions to account for complementarity outside the region. Conservation criteria based on the presence of seed region in an island of conservation is then incorporated by using groups of orthologous 3' UTR as input data. All of the above aims to efficiently reduce the false positive rates. However, the large amount of predictive targets for the let-7 miRNA family constitutes a complexity that can be difficult to interpret and explore. Since the accuracy of prediction of target sequences is low, it is difficult to identify bona fide gene targets regulated by miRNAs using Targetscan alone. Also such an approach will not yield genes indirectly regulated by miRNAs. Therefore we decided to focus on networks generated by Targetscan predictions rather than individual genes.

**Figure 8:**
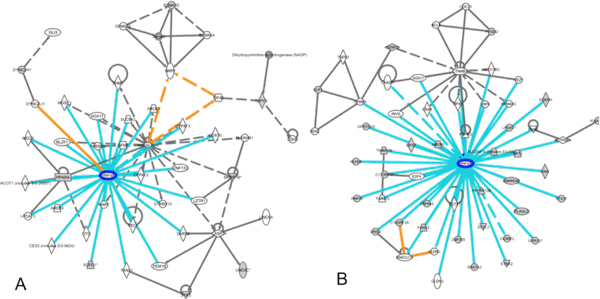
**HNF4A is a common hub for networks derived from alignment data and TargetScan predictions**. Gene interaction network on the left (A) is derived from the dataset of genes with overlapping regions corresponding to peaks from previous mapping. The other gene interaction networks (B) is derived from computationally predicted gene targets from TargetScan. Comparing both gene interaction network, similar topology was observed with HNF4A as a node amongst the interactions suggesting HNF4A as a possible downstream target for let-7 family miRNAs.

Our way of visualizing the roles of miRNA is via the concept of an integrated network emerging from the culmination of the interactions of the gene targets associated with the family of let-7 miRNAs. Such networks are tenuous but would serve sufficiently as a first order approximation. Networks generated using our previous genomic mapping alignment data were compared with the networks generated using TargetScan predictions. Examination of both networks (Figure 8) simultaneously revealed common nodes within these gene interaction networks. Hepatic nuclear factor 4 alpha (HNF4A) was found to be a common node in both networks making it a highly probable downstream target of indirect transcriptional regulation by let-7 family of miRNA. The expression of let-7 family of miRNAs was verified by quantitative real time PCR. let-7 b, let-7g, let-7f and let-7i miRNAs have threshold cycle (C_T_) values of 23.11, 22.85, 24.54 and 23.02 respectively compared to beta-Actin at a C_T _value of 15.5. We further compared the expression of Let 7 family of miRNA in Hep G2 cells. Hep G2 which is a liver cell line expresses high levels of HNF4A. let-7 miRNA was expressed at 5.7 fold higher levels in hES-MSC compared to HEPG2 cells, whereas HNF4A was undetectable in hES-MSC and very strongly expressed in HEPG2 cells (56,000 fold lower in hES-MSC {C_T _32.63} compared to HEPG2 cells{C_T _20.36}). Thus, a high level of expression of let-7 family of miRNA coincide with a low level of expression of HNF4A (e.g. hES_MSC) and vice versa (e.g. HEPG2).

**Figure 9:**
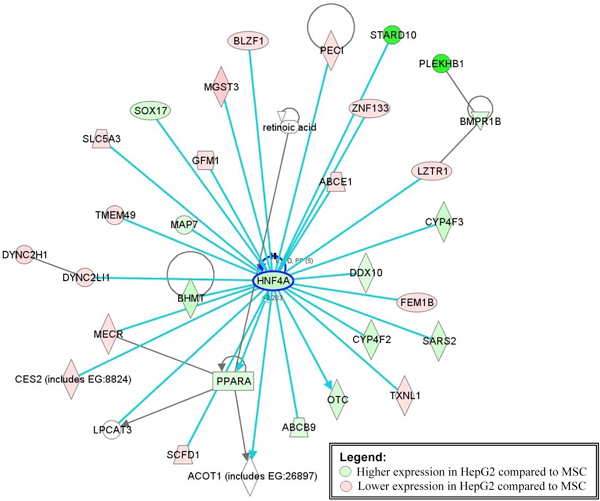
**Expression of genes in the HNF4A alignment network follows a similar pattern as HNF4A in hES-MSC and HEPG2 cells**. This figure shows the relative gene expression of genes from gene interaction network (A) in Figure 8 in HEPG2 cells compared to hES-MSC. It is interesting to note that most genes in this network are suppressed when let-7 family miRNAs are over-expressed (hES-MSC) and up-regulated when let-7 family gene expression goes down (HEPG2 cells).

To test the hypothesis that the network of genes surrounding HNF4A was controlled by let-7 family miRNA, we compared the expression of genes identified in the let-7 family alignment network in HEPG2 cells and hES-MSC. Figure 9 shows the relative gene expression of these genes in HEPG2 compared to hES-MSC. Most genes in this network show a higher expression in HEPG2 cells (let-7 family miRNA expression low) relative to hES-MSC (let-7 family miRNA expression high) providing further support to our hypothesis that let-7 family of miRNAs are regulating these genes.

## Discussion

We have developed a workflow for analyzing deep sequencing data focusing on miRNA. This workflow has revealed several trends in miRNA expression profile for hES-MSCs. The majority of sequenced transcripts were identified by mapping to known small RNA libraries and a considerable proportion of these were miRNAs. Several studies have showed that certain miRNAs are associated with specific stem cell types and those that are regulated during stem cell differentiation. Characterizing miRNA expression profile for the purpose of extracting biological function from sequencing data can be a challenge.

Raw sequencing data of our samples showed distinct distribution patterns across a large range of magnitude for transcripts counts. The necessity of a minimum threshold of transcript count for biological significance was addressed by adaptive thresholding exploiting KS statistics amongst biological replicates giving a lower bound value for transcript counts. Currently KS statistics are used primarily for comparing distributions and have been used in areas such as image processing for examining pixel distribution. In our study, we employed KS statistics to estimate the lower bound where distributions of transcripts peaks begin to differ. Using a transcript count of 32 as a lower bound, we were able to define a threshold for miRNA expression based on transcript abundance. Further comparisons (as shown in Figure 4) of top ranked genes between our sequenced data with microarray data showed that there is a considerable degree of overlap between the two especially in the intracellular samples. miRNAs from the extra-cellular samples form a subset of those expressed in the intracellular samples. Among the most abundantly expressed transcripts across both intra and extra-cellular environment (Tables 1 &2) the let-7 family of miRNAs was the only overexpressed family of known miRNAs.

Amongst other over expressed miRNAs, miR199b has transcription factors like SOX4 and has been shown to be involved in liver cancer and muscular dystrophy [10,11]. miR143 has recently been shown to cooperate with miR 145 to regulate plasticity and cell fate determination of smooth muscle cells via regulation of expression of transcription factors like Kruppel like factor 4 (Klf-4), myocardin and Elk-1 [12].

let-7 mutant phenotype studied in *C. elegans *has been associated with a lack of terminal differentiation and ongoing cell proliferation, both of which are characteristics of stem cells and cancer. let-7 targets include cell cycle regulators such as CDC25A and CDK6 [13]; promoters of growth including RAS and c-myc [14,15] and a number of early embryonic genes including HMGA2, Mlin-41 and IMP-1 [16,17]. Further, let-7 also targets Dicer [18,19] which is the protein responsible for miRNA maturation. Therefore it is possible that the let-7 family of miRNA acts as a master regulator of miRNA function. Since the let-7 family of miRNAs were abundantly expressed in MSC and given their central role in controlling cellular differentiation and miRNA regulation, we decided to focus on this family of miRNAs for further investigations.

miRNA mediated translational repression might play an important role in stem cell self-renewal. In our study, a high level of let-7 family of miRNA transcripts was predominant in both intra and extra cellular samples for our hES-MSC. This was true for other abundantly expressed miRNAs in the extra cellular space as well. We were unable to find miRNAs that were expressed only in the extra cellular space or were expressed more abundantly in the extra cellular space compared to the intra cellular space. This suggests that the process of secretion of miRNAs is most likely a passive one unlike that of proteins, where signal peptide containing proteins are transported out of the cell as soon as they are synthesized.

Moreover, the elevated presence of miRNAs in the extra cellular environment suggests a possible intercellular signaling mechanism. Recent literature has also supported that genetic exchange of mRNA and miRNA between cells can be accomplished through microvesicles transfer [7,8]. miRNAs like miR 21 [20], miR 133 and miR 30 [21] have been shown to play a role in cardiac remodelling in infracted hearts. Since miRNA can affect cardiac remodelling and these miRNAs are transported in micro vesicles by transplanted MSC (Lim SK personal communication), it is possible that transplanted MSC regulate remodelling of the surrounding myocardium through secreted miRNAs in addition to secreted cytokines and soluble factors.

HNF4A is a transcription factor that has also been shown to be essential for morphological and functional differentiation of hepatocyte and for liver morphogenesis [22]. HNF4A is also one of the physiological factors in the liver to activate apoB gene expression at the AF-1 site [23]. Elevated levels of apoB have been correlated with an increased risk of atherosclerosis and coronary heart disease [24]. HNF4A is also involved in the regulation of serum lipid levels and is linked to elevated serum cholesterol and triglyceride levels in Finnish combined familial hyperlipidemia patients [25]. However to date there is no evidence of miRNA regulation of HNF4A. None of the target prediction algorithms predict the regulation of HNF4A by let-7 family of miRNAs. There are no predicted miRNA binding sites in the untranslated or intronic regions of the HNF4A gene. However combining the networks generated by sequence alignment of expressed miRNAs and Targetscan, we predict that HNF4A is indirectly regulated by the let-7 family of miRNAs. We were able to confirm this indirectly by measuring the levels of let-7 family of miRNAs and HNF4A in undifferentiated MSC and HEPG2 cells. In undifferentiated MSC when let-7 miRNAs are highly expressed, expression of HNF4A is very low. Conversely in HEPG2 cells where a high level of HNF4A is expressed, we find very low expression of let-7 family miRNAs. Since HNF family of transcription factors have been reported to be upregulated in hepatocytes derived from adipose tissue MSC [26], it is possible that let-7 regulates HNF4A levels during this process. Since genes in the HNF4A alignment network also show a similar expression profile to HNF4A in HEPG2 and hES-MSC cells, it is possible that the let-7 family miRNA regulation of HNF4A is mediated through genes in this network. It is also interesting to note that there is no predicted miRNA target site in the UTR of HNF4A. Thus miRNA based regulation of HNF4A may be indirectly achieved through regulation of genes in the network that interact with HNF4A. It will be interesting to study the effect of Lin28 overexpression on differentiation of hepatocytes from hES-MSC since Lin 28 is a transcription factor that inhibits function of let-7 family miRNAs.

let-7f miRNA is known to exert pro-angiogenic effects [27]. Human MSC conditioned medium has been shown to reduce infarct size in patients with acute myocardial infraction [28]. Since MSC conditioned medium contains exosomes with let-7 family miRNAs and these let-7 family miRNAs may regulate HNF4A (based on our network and expression analysis), it is highly likely that MSC conditioned medium mediated reduction of infarct size is achieved by indirect regulation of HNF4A mediated by the let-7 family of miRNAs.

Thus, our study suggests the possibility of let-7 family of miRNAs indirectly regulating this particular transcription factor to achieve physiological changes. This is in line with recent paradigm where miRNAs regulate self-renewal and differentiation pathways of embryonic stem cells by forming an integral biological network with transcription factors [29].

## Conclusion

In conclusion, our study using a combination of different available tools to examine deep sequencing data by examining alignment, computer predictions, mathematical and network analysis has led us to a hypothesis that HNF4A is indirectly regulated by the let-7 family of miRNAs.

## Methods

### hES-MSC intracellular samples and microvesicles isolation from culture media

Human embryonic derived mesenchymal stem cells were used in this study. Two intracellular samples were obtained at passage 18 by lysing the collected cells. RNA was isolated from conditioned medium by adding three volumes of Trizol LS (Invitrogen) to one volume of conditioned medium and completing the extraction according to the manufacturer's protocol. Total RNA and small RNAs from MSC were purified using Trizol (AppliedBiosystems) and mirVana™ miRNA Isolation Kit (AppliedBiosystems), respectively. RNA was quantiated using Quanti-T™ RiboGreen^® ^RNA Assay Kit (Invitrogen). In a separate experiment, the conditioned medium was treated with RNase, RNA extracted and compared to RNA extracted from untreated conditioned medium. No difference was observed in the RNA profile from the RNase treated and untreated samples suggesting that the RNA in the conditioned medium was contained within the exosomes (data not shown). Resulting samples were sent to Illumina Sequencing facilities for Deep Sequencing. The intracellular samples were further gel purified to only include transcripts between 18-35 nucleotides long. This is a step in the standard sample purification protocol for Deep Sequencing. This step was omitted in the samples of hES-MSC conditioned medium profiling the extra cellular small RNAs. However since all miRNAs are in the 18-35 nucleotide range this step has no effect on the data analysis described here which considered only known miRNAs.

### Extracting biologically significant miRNA profiles from raw Deep Sequencing transcripts counts between intra and extra-cellular samples through data visualization tools and plots

Deep sequencing of small RNAs was performed using the Illumina/Solexa platform (sequencing depth 5.5 million reads) which produces individual sequence reads with base quality scores. Identical sequences were counted and removed. The resulting dataset consisted of unique sequences with the associated read counts. Prior to further analysis as show in the workflow (Figure 1), the adaptor sequences were trimmed computationally. Resulting sequences were mapped against known small RNA libraries to identify sequences originating from sources such as rRNA, tRNA, snRNA and snoRNA. Focusing primarily on the dataset consisting of only known human miRNAs, only small RNA sequences that align to mirBASE were retained.

These sequences were aligned to the human genome using Seqmap [30] with a 3 nucleotide mis-match condition. We arrived at the 3 nucleotide mismatch condition after looking at the distribution of the aligned sequences at 1, 2, 3 and 4 nucleotide mismatch. The distribution changed significantly after 3 nucleotide mismatch. Therefore we concluded that this was the maximum allowable mismatch for aligning the sequenced transcripts. The Illumina ELAND alignment tool which yields similar results as Seqmap also uses a 3 nucleotide mismatch for aligning RNA seq data. The alignment results were visualized using UCSC genome browser [31]. Genomic graphs depict the spatial distribution across the genome and are characterized by peaks along the genome loci corresponding to transcript count that align to that region. The magnitude of each peak is a reflection of the number of transcripts. Histograms were used to describe the frequency of occurrence of theses peaks for further analysis.

### Determining minimum threshold count of biological significance

The process of quantifying the number and magnitude of peaks due to each miRNA necessitates a threshold level to differentiate miRNAs of biological significance from background noise. We quantified a threshold value by deploying a strategy via applying Kolomogorov-Smirnov (KS) tests to our data iteratively.

Kolomogorov-Smirnov test is a form of minimum distance estimation. In this aspect, we used it as a nonparametric test of equality for comparing distributions from our biological replicates. The KS test statistics quantifies the distance between the cumulative distributions of our replicates and thus can be viewed as a measure of agreement between them. The threshold number of transcripts was changed with each application of the KS test and the KS statistics were recorded. The final threshold count is the one which corresponds to the first instance of a minimum point in the KS statistics value.

This condition can be thought of as the first instance when the distributions of miRNA amongst replicates began to be similar. Such an approach can be thought of as a form of adaptive thresholding. The key parameter in a thresholding process is the choice of the threshold value. Usually, this value is arbitrary chosen. Our approach computes this value automatically considering the replicates of our experiments. Threshold values can thus be uniquely generated for each experimental protocol or cell type to differentiate biological significance from background noise. Observation of the distributions of transcripts from deep sequencing suggested the presence of different distributions within each of the intracellular samples. Application of KS statistics to our data ensures that further interpretations are based on reproducible parts of the overall distributions. KS statistics also provide a lower threshold boundary which provided a quantitative justification for selecting candidates for detailed analysis in later stages of the workflow [32].

Having determined the threshold, we then proceeded to characterize the salient features of miRNA expression profiles by ranking the top miRNAs that accounted for the large magnitude peaks observed in the genomic graphs. The top 100 ranked miRNAs were compared to a similar list derived from a miRNA microarray experiment of the same samples as a measure of consistency.

### Network Analysis for predicting indirect miRNA gene Targets

TargetScan [33] was used to predict gene targets for the let-7 family of miRNAs. An alternative representation of the TargetScan results was used in our study where the hypothesis is that biological pathways and networks rather than the individual genes are driving development of the range of phenotypes observed. It is such networks and interactions that we would like to understand. Using such framework to conceptualize predicted miRNA gene targets from TargetScan, the targets for the let-7 family of miRNA were subjected to pathway exploration using the Ingenuity Pathway Analysis (Ingenuity^® ^Systems, http://www.ingenuity.com) software. Using Ingenuity Pathway Analysis and accompanying interaction database, top ranking interaction networks were generated.

Our approach in reducing complexity of gene interactions networks uses the integration of the data from different sources. This was done by comparing the network generated based on our data from mapping the respective peaks along genomic location with those derived from the TargetScan prediction. Since Targetscan over predicts miRNA targets, we compared networks generated with genes predicted by Targetscan with networks generated by genes from our alignment data. We hoped to extract biological significance from the expressed miRNAs by sieving for common nodes in these networks of predicted miRNA targets. These common nodes of gene targets strongly suggest a down stream target being regulated by the respective miRNAs.

### Measurements of expression levels of let-7 family of miRNAs and HNF4A by quantitative real time reverse transcriptase polymerase chain reaction (qRT-PCR)

RNA from cultured hES-MSC and HEPG2 cells was extracted using Trizol reagent (Invitrogen, Carlsbad, CA). qRT-PCR was performed using Taqman assays according to manufacturer's instructions. For calculating relative fold change values, the C_T _values were normalized to U6 miRNA as internal control for miRNA and GAPDH for HNF4A. We chose U6 as the internal control because it is similar in length and structure to known miRNAs.

Further we also verified the prediction that let-7 family miRNAs regulate the network of 50 genes, by examining the expression profiles of these genes in MSC and HEPG2 cells. The mRNA expression for 12 hES-MSC samples was profiled by our lab using the Illumina Ref8 v3 Expression BeadChip (data not shown). The mRNA expression profiles for two HepG2 control samples were obtained from ArrayExpress (ID: E-MEXP-1213). We chose these samples because the mRNA profiling was also performed on Illumina microarrays (HumanWG-6 v3), allowing for more comparable expression values. Expression values for the 50 genes identified in the HNF4A alignment network were extracted for each sample. The expression values were normalized by dividing them by the level of expression of a housekeeping gene, glyceraldehyde 3-phosphate dehydrogenase (GAPDH) in each sample. Then, the normalized expression value for each gene was averaged across the 12 hES-MSC samples, and across 2 HEPG2 samples. A ratio of averaged normalized gene expression value for each gene was generated by comparing expression in HEPG2 to hES-MSC cells. The relative expression for these 50 genes was visualized using Ingenuity Pathway Analysis.

## Competing interests

The authors declare that they have no competing interests.

## Authors' contributions

LSK and CTS cultivated the hES-MSC samples and prepared them for sequencing. Conception and development of analysis strategies were contextualized by VK, BT, WK and VT. VK and WK provides statistical analysis of data. WK analysed results and drafted the manuscript. BT carried out the laboratory experiments. QYL analyzed the expression data of the 50 genes in HNF4A alignment network for HEPG2 cells and hES-MSC. VT conceived the study, and participated in its design and coordination and together with VK revised the manuscript.
